# Targeted Delivery of Chlorin e6 *via* Redox Sensitive Diselenide-Containing Micelles for Improved Photodynamic Therapy in Cluster of Differentiation 44-Overexpressing Breast Cancer

**DOI:** 10.3389/fphar.2019.00369

**Published:** 2019-04-16

**Authors:** Chan Feng, Donglei Zhu, Lv Chen, Yonglin Lu, Jie Liu, Na Yoon Kim, Shujing Liang, Xia Zhang, Yun Lin, Yabin Ma, Chunyan Dong

**Affiliations:** ^1^ Cancer Center, Shanghai East Hospital, Tongji University, Shanghai, China; ^2^ Department of Chemical Engineering, Northeastern University, Boston, MA, United States; ^3^ Pharmacy Department, Shanghai East Hospital, Tongji University, Shanghai, China

**Keywords:** chlorin e6, redox sensitive, diselenide, photodynamic therapy, cluster of differentiation 44, targeted delivery, breast cancer

## Abstract

The off-target activation of photosensitizers is one of the most well-known obstacles to effective photodynamic therapy (PDT). The selected activation of photosensitizers in cancer cells is highly desired to overcome this problem. We developed a strategy that enabled diselenide bonds to link hyaluronic acid (HA) and photosensitizer chlorin e6 (Ce6) to assemble the micelles (HA-sese-Ce6 NPs) that can target cancer and achieve a redox responsive release of drugs to enhance the PDT efficiency in breast cancer. The HA was used to form a hydrophilic shell that can target cluster of differentiation 44 (CD44) on the cancer cells. The selenium-containing core is easily dissembled in a redox environment to release Ce6. The triggered release of Ce6 in a redox condition and the positive feedback release by activated Ce6 were observed *in vitro*. In cytotoxicity assays and *in vitro* cellular uptake assays, the increased PDT efficiency and targeted internalization of HA-sese-Ce6 NPs in the cells were verified, compared to a free Ce6 treated group. Similar results were showed in the therapeutic study and *in vivo* fluorescence imaging in an orthotopic mammary fat pad tumor model. In addition, a significant inhibition of metastasis was found after the HA-sese-Ce6 NPs treatment. In general, this study promises an ingenious and easy strategy for improved PDT efficiency.

## Introduction

Photodynamic therapy (PDT) is a promising noninvasive, localized therapeutic method that has a variety of advantages for cancer treatment, especially for tumors located close to the skin such as breast cancer (Agostinis et al., 2011; Wang et al., 2018). One of the most widely used photosensitizers in PDT is chlorin e6 (Ce6), a second-generation photosensitizer with high efficacy and low dark toxicity (Martynenko et al., 2015; Du et al., 2016). The therapeutic effect of PDT is based on the activation of photosensitizers. When photosensitizers are exposed to a certain wavelength of light, they release singlet oxygen (^1^O_2_) that can kill cancer cells. However, there are limitations to this therapy. Due to the short half-life (<40 ns) of ^1^O_2_ during PDT, each ^1^O_2_ molecule can only have therapeutic effects in the area of diameter less than 20 nm (Li et al., 2017; Zhang et al., 2018). This makes the precise delivery of photosensitizers highly desirable. Moreover, nonspecific activation of photosensitizers will cause potential cytotoxicity in normal tissue. Thus, selected delivery of photosensitizers to tumor sites is high desired. Beyond the previously mentioned challenges, the vast majority of photosensitizers, including Ce6, have a poor water solubility, which leads to undesired pharmacokinetics (Liu et al., 2017).

In the past few decades, various advanced nanoscale drug delivery systems have been developed to optimize pharmacokinetics by selectively delivering hydrophobic drugs and photosensitizers to achieve better efficacy and less off-target side effects in cancer treatment (Ji et al., 2015, 2017; Ouyang et al., 2018a,b). In addition, smart drug release has been extensively investigated for precise drug release (Ji et al., 2016a,b). By utilizing stimuli-responsive linkages, drug release can be triggered by various specific tumor biological/endogenous stimuli, such as pH (Webb et al., 2011), redox (Go and Jones, 2008), hypoxia (Brown and Wilson, 2004), or enzymes (de la Rica et al., 2012). However, a complicated stimuli-responsive design leads to a larger proportion of drug carrier and limited drug loading capacities. Nowadays, the clinical translation of nanoparticle-based drug delivery has several limitations. One of most controversial problems is the increasing cost of biosafety due to the risk of using synthetic materials in drug carriers. Therefore, designing drug carriers that are simple and made of biocompatible materials is highly desired.

Selenium, one of the essential dietary elements in higher animals, plays an important role in cell growth and functions (Fang et al., 2018). A few studies even reported anticancer activity of selenium (Yu et al., 2015; Baskar et al., 2018). Interestingly, the relatively low electronegativity and large atomic radius give selenium unique chemical properties, such as high reactivity and sensitivity (Xia et al., 2016, 2018b; Sun et al., 2017). Due to their high sensitivity to both oxidation and reduction, diselenide-containing polymers have been gaining attention as attractive drug delivery candidates that can perform controlled drug release in tumor microenvironment with rich redox stimuli (Ma et al., 2010; Gong et al., 2017; Zhou et al., 2017, 2018). Another characteristic of diselenide bonds that is particularly advantageous in PDT drug delivery is their sensitivity to both ^1^O_2_ and 600 nm or higher wavelength light (Xia et al., 2016; Sun et al., 2017). If diselenide bonds in a drug delivery system could be cleaved by ^1^O_2_ and a laser stronger than 600 nm and release drug molecules that produce more ^1^O_2_, the positive feedback on drug release is theoretically possible.

Hyaluronic acid (HA) is a natural anionic hydrophilic polysaccharide in the human body and is especially over-expressed in the tumor matrix (Choi et al., 2011; Agrawal et al., 2018; Huang and Huang, 2018a). In many cancers of epithelial origin, such as breast cancer, cluster of differentiation 44 (CD44) is a main up-regulated HA receptor on the cancer cell surface (Huang and Huang, 2018b; Liao et al., 2018; Maudens et al., 2018). HA can regulate cancer cell proliferation and migration *via* CD44 (Safdar et al., 2017; Tian et al., 2018). Thus, HA has gained attention as a promising cancer targeting ligand for anti-cancer drug delivery. In addition, HA has high water solubility, desirable biocompatibility, biodegradability, and nonimmunogenicity and can be easily functionalized (Choi et al., 2011; Xia et al., 2018a). Many HA-drug conjugates and HA-based micelles have been developed.

**SCHEME 1:**
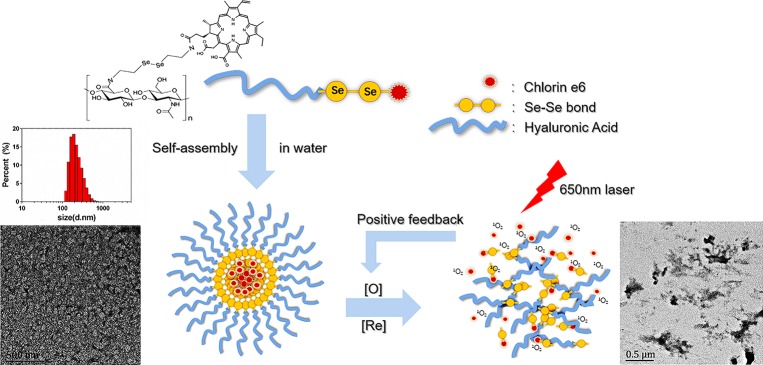
Scheme of self-assembling HA-sese-Ce6 micelles, redox sensitive Ce6 release, and positive feedback loop that triggers more Ce6 release.

**SCHEME 2:**
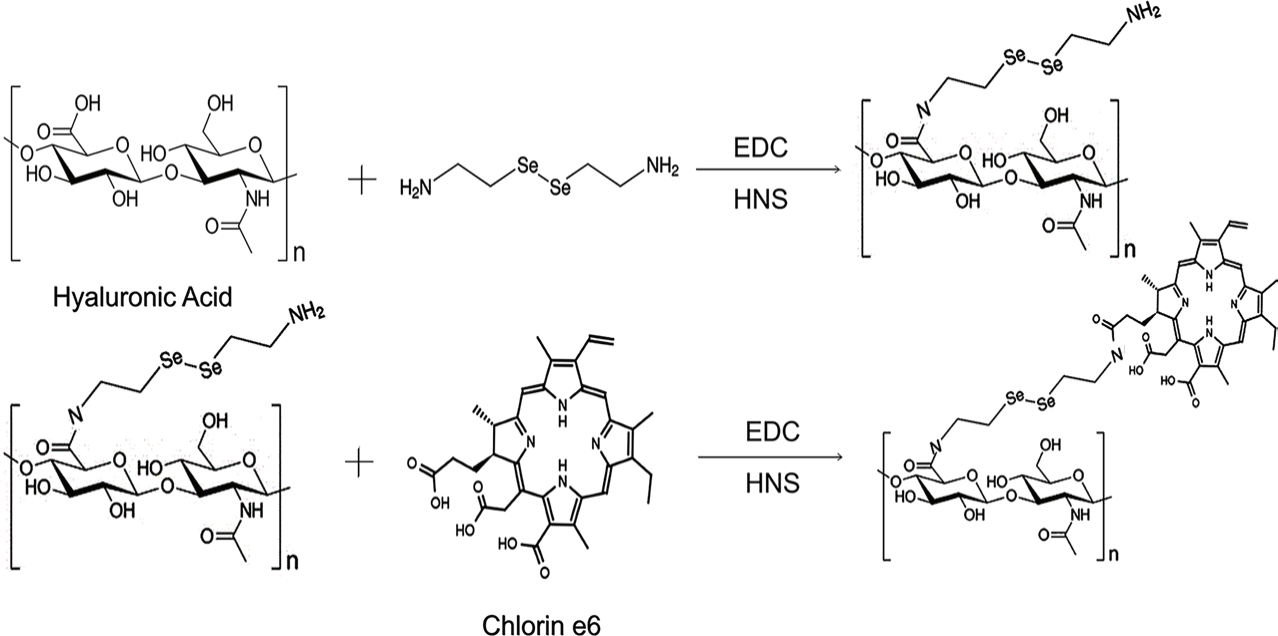
The synthetic process of HA-sese-Ce6.

Here, we developed a minimalist photosensitizer delivery system, incorporating diselenide bonds into a self-assembled micelle. HA was chosen as the hydrophilic shell and grafted onto a hydrophobic core, Ce6, *via* diselenide bonds. The amphiphilic hyaluronic acid-chlorin e6 (HA-SeSe-Ce6) polymers were synthesized and formed micelles by self-assembly (Schemes 1, 2). In our *in vitro* study, the redox-responsive and positive feedback modulated release of Ce6 was observed when the diselenide bonds were cleaved in redox condition and in the presence of ^1^O_2_. The PDT efficacy was investigated in a breast cancer cell line and orthotopic mammary fat pad tumor model.

## Materials and Methods

### Materials

Chlorin e6 was purchased from J&K Scientific, Ltd. 2-(N-morpholino), Selenocystamine dihydrochloride (C_4_H_12_N_2_Se_2_·2HCl), N-hydroxysuccinimide (NHS, 98%), and 1-ethyl-3-(3(dimethylamino)propyl) carbodiimide hydrochloride (EDC·HCl, 98.5%) were obtained from Sigma-Aldrich (Shanghai). Hyaluronic acid (mW ≈ 20 k) was purchased from Ruixi biotechnology Co., Ltd. Singlet oxygen sensor green (SOSG) was obtained from Life Technologies. Hydrogen peroxide solution (H_2_O_2_, 30 wt. % in H_2_O), glutathione (GSH, 98%), dimethyl sulfoxide (DMSO, 99.9%), and NaOH (AR, 96%) were obtained from Aladdin Chemistry Co., Ltd. Dulbecco’s modified eagle medium (DMEM), penicillin-streptomycin, fetal bovine serum (FBS), and trypsin were purchased from Gibco Invitrogen Corp. CCK-8 and 4, 6-diamidino-2-phenylindole (DAPI) were obtained from Beyotime Institute of Biotechnology. Paraformaldehyde (4%) was obtained from DingGuo Biotech. Co., Ltd. One step TUNEL apoptosis assay kit was purchased in Beyotime biotechnology Co., Ltd.

### Methods

#### Synthesis of HA-sese-Ce6 Micelles

In a small glass bottle, 20-mg HA, 10-mg C_4_H_12_N_2_Se_2_·2HCl, 5.75-mg EDC·HCl, 3.45-mg NHS, and 10-ml PBS (pH 7.4) were mixed and stirred at room temperature for 2 h. Ce6 was dissolved in 20-mg/ml DMSO solution. Then, 0.9 ml of Ce6 liquid, 5.75 mg of EDC·HCl, and 3.45 mg of NHS were added dropwise to the mixed liquid and stirred for 4 h. The solution was transferred to a 100 kDa mol. Cutoff centrifugal ultrafiltration tube (Pall Corporation, USA) was centrifuged at 4500 rpm at 25°C for 20 min. It was washed three times with deionized water to remove PBS, unassembled HA, C_4_H_12_N_2_Se_2_·2HCl, and Ce6.

#### Characterization of HA-sese-Ce6 Micelles

Fluorescence spectra were performed on a Hitachi F2500 luminescence spectrometer. Ultraviolet-visible (UV) spectra were recorded on a UV spectrophotometer (Varian). The size distribution of the micelles was characterized by Nano-ZS 90 Nanosizer (Malvern Instruments, Worcestershire, UK) *via* dynamic light-scattering analysis. The morphology of micelle was studied *via* high-resolution transmission electron microscopy (HRTEM, FEI Tccnai G2 F20 S-Twin). About 1% uranyl acetate was used for negative staining.

#### Chlorin e6 Release Behavior of HA-sese-Ce6 Micelles *in vitro*

HA-sese-Ce6 (containing 5 mg of Ce6) was dissolved in 5 ml of PBS buffer (10 mM, pH 7.4). The solution was transferred to five dialysis tubes (1 ml each) that were immersed in 200 ml of PBS buffer, 200 ml PBS buffer with 10 Mm GSH, 200 ml PBS buffer with 100 Mm GSH, 200 ml PBS buffer with 1 Mm H_2_O_2_, and 200 ml PBS buffer with 10 Mm H_2_O_2_ at 37°C, stirred at 200 r min^−1^. To evaluate the positive release of Ce6 from HA-sese-Ce6 micelles, the 1 ml of HA-sese-Ce6 (containing 1 mg Ce6) in PBS buffer was exposed to a 650-nm laser that has the light intensity of 20 mW/cm^2^ for 10 min. Then, the solution was transferred to a dialysis tube and immersed in 200 ml of PBS buffer. At predetermined time points, 1 ml of the buffer solution outside the dialysis tube was taken out, and the Ce6 release was measured on a UV spectrophotometer at 404 nm. Ce6 release curves of six groups were drawn.

#### Measurement of Singlet Oxygen (^1^O_2_) Generation

One milliliter of the buffer solution outside the dialysis tube was taken out from both 200 ml of PBS buffer with 10 Mm GSH and 200 ml of PBS buffer with 1 Mm H_2_O_2_, which were previously described in method 3. To measure the ^1^O_2_ generated from activated Ce6, ^1^O_2_ detecting reagent SOSG was added to the solution. The final concentration of SOSG in the solution was 1 μM. The fluorescence emission spectra were recorded from 490 to 700 nm, and the excitation wavelength was 488 nm.

#### *In vitro* Cellular Uptake Assay

The mouse breast cancer cell line 4T1 cells were purchased from ATCC. 4T1 cells were cultured in DMEM containing 10% FBS. The cell cultures were maintained in 5% carbon dioxide at 37°C. To investigate the targeted uptake of HA-sese-Ce6 micelles by 4T1 cells, the cellular uptake was analyzed by confocal laser scanning microscopy (CLSM) and flow cytometry (FCM). 4T1 cells (1 × 10^5^ cells/well) were cultivated in confocal dishes for 24 h. Then, the cells were treated with serum-free DMEM containing Ce6 and HA-sese-Ce6 micelles (Ce6 concentration of 2 μM). After 4 h, the medium was removed. The cells were washed with PBS and fixed with paraformaldehyde (4%) for 10 min. Afterward, cells were stained with DAPI for 5 min and washed three times. The dishes were measured by confocal laser scanning microscopy (Leica TCS SP5 II, Germany). 4T1 cells (1 × 10^5^ cells/well) were seeded on six wells and cultivated for 24 h. The medium in the dishes was removed, and Ce6 and HA-sese-Ce6 micelles in serum-free DMEM medium were added. Then cells were harvested twice, 4 and 12 h after incubation, and resuspended in 400 μl of PBS and were analyzed through flow cytometry.

#### *In vitro* Phototoxicity Test

In 96-well-plates, 1 × 10^4^ cells/well 4T1 cells were planted and treated with different concentrations (0.25, 0.5, 1, and 2 μM) of Ce6 and HA-sese-Ce6 micelles in serum-free DMEM. In the control group, the same volume of serum-free DMEM was added. After the 24-h incubation period, the medium in the plates was removed, and the fresh medium was added. Half of the cells were exposed to 650 nm laser (20 mW/cm^2^) for 5 min, while the other half was cultured in the dark. After the 24-h incubation period, 10 μl of cck8 reagent was added to each well to measure cell proliferation. Three hours later, the absorbance at 450 nm was measured by the plate reader.

#### *In vivo* Fluorescence Imaging

This study was carried out in accordance with the recommendations of Tongji University Animal Ethics guidelines. The protocol was approved by Tongji University Animal Ethics Committee.

The 5 × 10^5^ 4T1 cells were injected subcutaneously into 5-week-old female BALB/c mice. After 2 weeks, tumor tissues were excised and cut into 1 × 1 mm^2^ tissue blocks to plant in the left mammary fat pad of 5-week-old female BALB/c mice. When the tumor size was large enough, Ce6 and HA-sese-Ce6 micelles were injected into the tail vein of the mice bearing a 4T1 tumor. Fluorescence imaging was performed by a Night OWL LB 983 *in vivo* imaging system 1 and 2 h after the injection.

#### *In vivo* Photodynamic Therapy

Tumor tissues were planted in the mammary fat pad of 5-week-old female BACB/c mice on day 0. On day 13, the volume of the tumors reached 500 mm^3^, and the mice bearing a tumor in the mammary fat pad were randomly assigned to three groups (*N* = 6). These mice were treated with Ce6 and HA-sese-Ce6 micelles (Ce6 dose of 2.5 mg/kg) in PBS every 2 days over the course of 10 days. Two hours after injection, tumors were exposed to 650 nm laser at the intensity of 20 mW/cm^2^ for 30 min. The body weight and tumor size were recorded before each injection, and tumor volumes were calculated by the following formula:

$$\mathrm{Tumor}\;\mathrm{volume}=\frac{\mathrm{Length}\times {\mathrm{Width}}^{2}}{2}$$

On day 30, one of mice was randomly picked from each group, and the major organs (heart, liver, spleen, lungs, and kidneys) and tumors were harvested. The collected samples were fixed in 4% paraformaldehyde overnight, dehydrated in graded ethanol solution, and embedded in paraffin. Paraffin sections were prepared to perform the H&E and TUNEL staining. The percent survival of mice (*N* = 5) was recorded until day 40. Tumors weights were recorded upon the death of mice.

### Statistical Analysis

All experiments were performed in three independent experiments. One-way single factorial analysis of variance (ANOVA) was used for determining the statistical significance of the data, which were expressed as *p* * **≤** 0.05, ** **≤** 0.01, *** **≤** 0.001.

## Results and Discussion

### Synthesis of HA-sese-Ce6 Micelles

The chemical structure and synthetic process of HA-sese-NH2 and HA-sese-Ce6 were shown in Scheme 2. HA-sese-NH2 was prepared by conjugating HA (mW ≈ 20 k) to selenocystamine dihydrochloride (C_4_H_12_N_2_Se_2_·2HCl) (molar mass proportion 1:1) *via* amino-carboxyl reaction. Subsequently, Ce6 was conjugated to the terminal amino group of HA-sese-NH2 *via* amino-carboxyl reaction. Due to its amphiphilic character, HA-sese-Ce6 can form micelles by self-assembly in water. As shown in Scheme 1, HA acts as a hydrophilic coat, and Ce6 acts as a hydrophobic core of the micelle.

The characteristics of HA-sese-Ce6 micelles were analyzed. The hydrodynamic diameter of the micelles was measured *via* DLS. The diameter was 250 nm, and the size had a narrow distribution. The TEM pictures showed the spherical shapes of the micelles. As the samples were dried during the TEM analysis, the size measured in the TEM analysis was smaller than that from the DLS analysis.

### *In vitro* Redox Sensitivity of HA-sese-Ce6 Micelles

**Figure 1:**
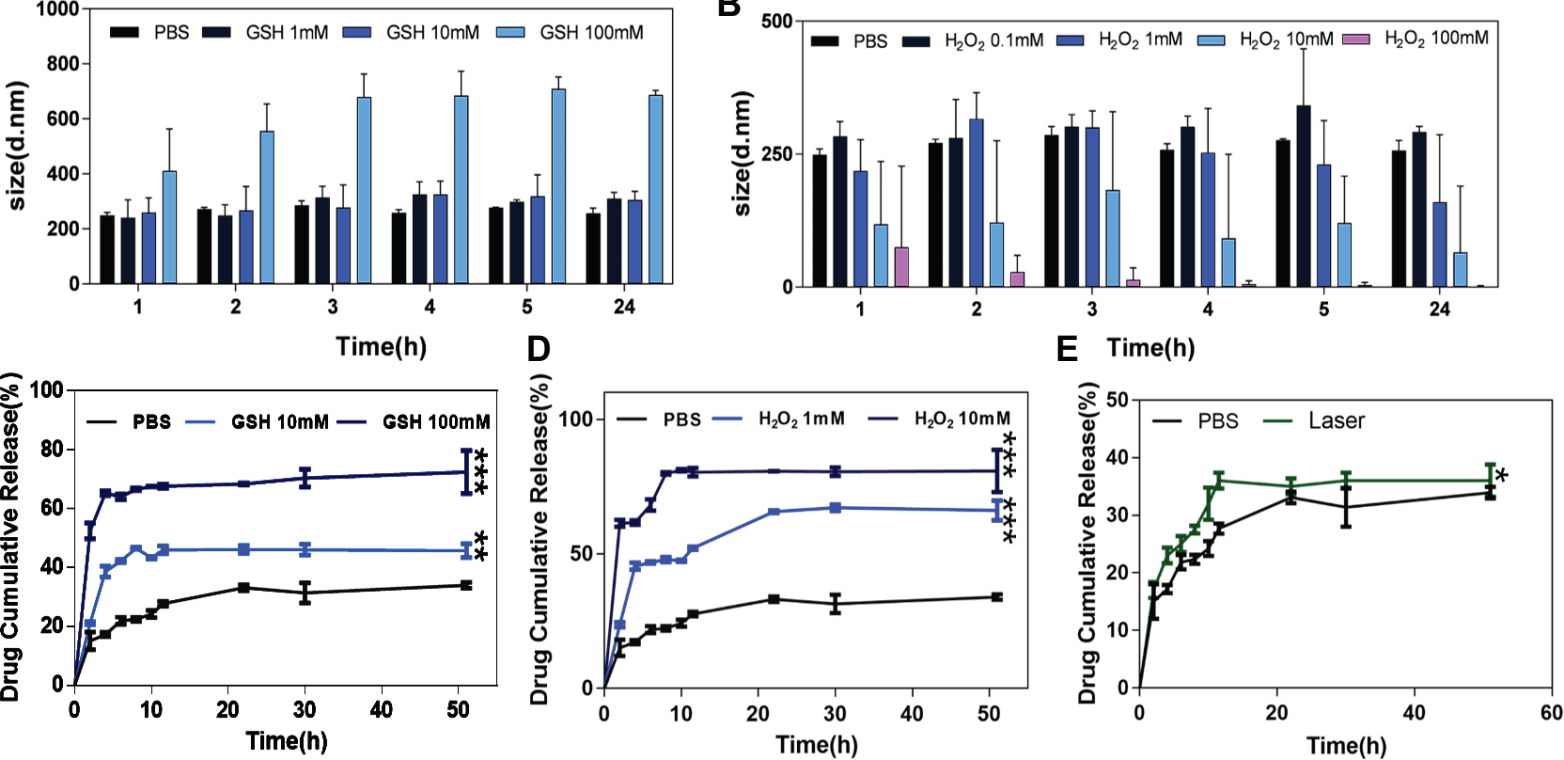
The size change and Ce6 release behavior of HA-sese-Ce6 micelles in different concentrations of GSH and H_2_O_2_. **(A)** The size of HA-sese-Ce6 micelles in PBS, 1 mM GSH, 10 mM GSH, 100 mM GSH, respectively. **(B)** The size of HA-sese-Ce6 micelles in PBS, 0.1 mM H_2_O_2_, 1 mM H_2_O_2_, 10 mM H_2_O_2_, 100 mM H_2_O_2_, respectively. **(C)** The Ce6 release of HA-sese-Ce6 micelles in PBS, 10 mM GSH, 100 mM GSH. **(D)** The Ce6 release of HA-sese-Ce6 micelles in PBS, 1 mM H_2_O_2_, 10 mM H_2_O_2_. **(E)** The Ce6 release of HA-sese-Ce6 when exposed to a 650 nm laser (20 mW/cm^2^) and PBS without laser treatment (the control group). The data are mean ± SD, *p* * **≤** 0.05, ** **≤** 0.01, *** **≤** 0.001 vs. the PBS.

HA-sese-Ce6 micelles were designed to be redox sensitive due to their diselenide component. The bond between diselenide breaks when it is exposed to the redox environment. To demonstrate the redox sensitivity of HA-sese-Ce6 micelles, they were treated with different concentrations of GSH and H_2_O_2_. The size changes of these micelles were recorded at predetermined time points. As shown in Figure 1A, the size of micelles tends to be larger when they were treated with higher concentration of GSH. A slight increase in size was observed between the micelles treated with 1 mM GSH and the micelles treated with 10 mM GSH. However, the micelles treated with 100 mM GSH showed a dramatic increase in size.

The size of the micelles treated with different concentrations of H_2_O_2_ (0.1, 1, 10, and 100 mM) were analyzed. The size of micelles treated with 0.1, 1, and 10 mM of H_2_O_2_^,^ increased and then decreased over time (Figure 1B). The micelles treated with 100 mM GSH had the smallest diameter, and the size consistently decreased over time. Our results could be explained by the effect the diselenide bond cleavages on the size of the micelles. Fewer cleavages of diselenide bonds would cause increase in size, whereas more cleavages of diselenide bonds would cause decrease in size, and redox sensitivity of HA-sese-Ce6 micelles can be demonstrated by the size changes in the reducing and oxidizing conditions.

### Chlorin e6 Release Behavior of HA-sese-Ce6 Micelles *in vitro*

To further evaluate the redox sensitivity of HA-sese-Ce6 micelles, the Ce6 release behavior was measured in different concentrations of GSH and H_2_O_2_ at 37°C. The results of GSH treated groups were shown in Figure 1C. Overall, the cumulative Ce6 release increased as the concentration of GSH increased. In the control group, which was treated with PBS, the cumulative release of Ce6 reached a plateau at 30% in 20 h. This could be explained by the physical adsorption effect of micelles on free Ce6. In the group treated with 10 mM GSH, the cumulative release of Ce6 reached a higher plateau at 45% in 11 h, while the group treated with 10 mM GSH reached the higher plateau 65% in 4 h.

As shown in Figure 1D, the micelle groups treated with H_2_O_2_ generally showed more Ce6 release than those treated with GSH. In the group treated with 100 mM H_2_O_2_, the cumulative release of Ce6 reached a highest plateau at 80%, which is higher than the highest plateau (65%) in the GSH treated group.

**Figure 2:**
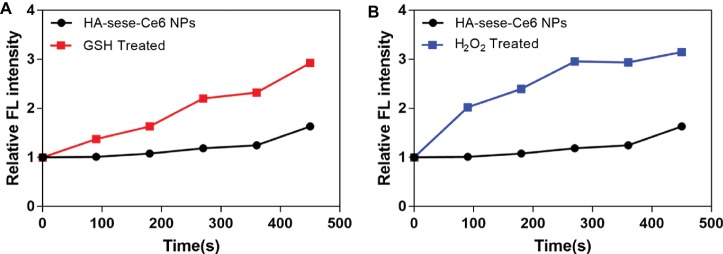
**(A)** Singlet oxygen generation by the released Ce6 from the micelles treated with GSH. **(B)** Singlet oxygen generation by the released Ce6 from the micelles treated with H_2_O_2_. The PBS treated group served as the control group. The data are mean ± SD, *p* *** **≤** 0.001 vs. the PBS.

As shown in Figure 2, increased singlet oxygen generation was observed in the micelle groups that were treated with GSH and H_2_O_2_. After Ce6 is released from HA-sese-Ce6 micelles due to redox stimuli, Ce6 gets activated and produces more singlet oxygen. This causes a positive feedback on the release of Ce6 because singlet oxygen triggers HA-sese-Ce6 micelles to release more Ce6. The results confirm the high sensitivity of HA-sese-Ce6 micelles to both oxidation and reduction. This suggests that HA-sese-Ce6 micelles would achieve smart drug release in tumor tissues with rich redox stimuli. As shown in Figure 1E, the higher drug release was observed when the micelles were treated with a laser. This could be explained by the indirect effect of the singlet oxygen generated from the activated Ce6 and direct effect of the 650 nm laser.

### Targeted Cellular Uptake and *in vitro* Cytotoxicity

**Figure 3:**
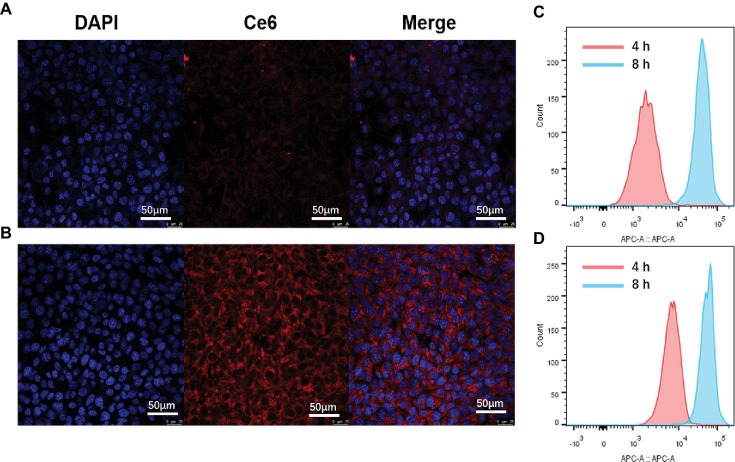
Cellular uptake of HA-sese-Ce6 micelles and free Ce6. CLSM micrographs. **(A)** cells treated with free Ce6 for 4 h, **(B)** cells treated with HA-sese-Ce6 micelles FCM profiles, **(C)** cells treated with free Ce6 for 4 and 8 h, and **(D)** cells treated with HA-sese-Ce6 micelles for 4 and 8 h.

Targeted cellular uptake of HA-sese-Ce6 micelles by cancer cells was investigated by CLSM and FCM. 4T1 cancer cells were treated with free Ce6 and HA-sese-Ce6 micelles for 4 and 8 h, respectively. The CLSM analysis was shown in Figures 3A,B. The stronger fluorescence was observed in the cells treated with HA-sese-Ce6 micelles, which indicates markedly higher intracellular uptake of HA-sese-Ce6 micelles when compared to free Ce6. As shown in Figures 3C,D, the FCM analysis showed the same results. The number of cells that internalized Ce6 was higher in the HA-sese-Ce6 micelles treated group than the free Ce6 treated group.

**Figure 4:**
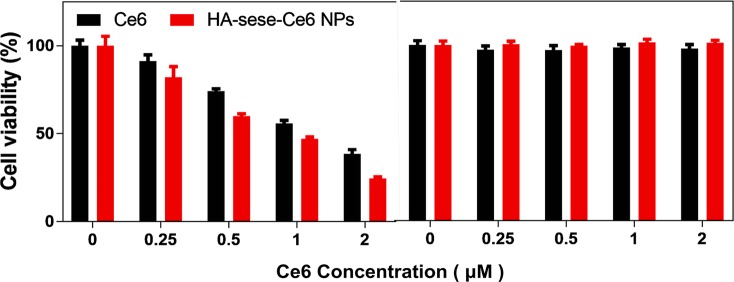
Phototoxicity of 4T1 cancer cells treated with free Ce6 and HA-sese-Ce6 micelles, at equivalent concentrations (0.25–2 μM Ce6). (**Left**) Dark toxicity of 4T1 cancer cells treated with free Ce6 and HA-sese-Ce6 micelles, at equivalent concentrations (0.25–2 μM Ce6) (**Right**).

To evaluate the anticancer efficacy of HA-sese-Ce6 micelles, 4T1 cancer cells were seeded in 96-well plants and treated with free Ce6 or HA-sese-Ce6 micelles. After the 24-h incubation, half the cells were exposed to a 650-nm laser (20 mW/cm^2^) for 5 min, and the other half of the cells were kept in the dark as a control. As the data shown in Figure 4, the cytotoxicity significantly increased as the Ce6 concentration increases in both groups that had the laser treatment. The cells treated with HA-sese-Ce6 micelles exhibited lower cell viability than free Ce6 treated cells at all concentrations. The targeted redox responsive delivery of HA-sese-Ce6 might be the explanation for lower viability of cancer cells. Both free Ce6 and HA-sese-Ce6 micelles treated cells without the laser exposure exhibited no significant toxicity.

### *In vivo* HA-sese-Ce6 Micelles Biodistribution in Breast Cancer Bearing Mice

**Figure 5:**
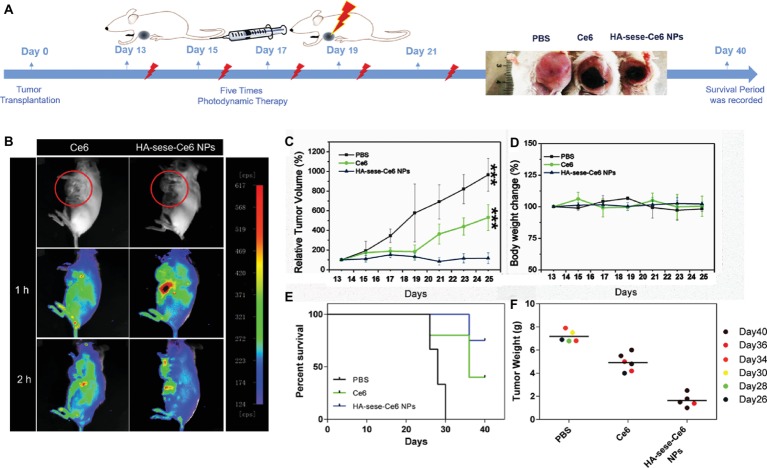
**(A)** Photodynamic therapy scheme in a breast tumor animal model. **(B)** *In vivo* fluorescence imaging 1 and 2 h after the injections of free Ce6 and HA-sese-Ce6 micelles, respectively red circles represent tumor regions. **(C)** Tumor growth curves of mice in the free Ce6 and HA-sese-Ce6 micelles treated groups, and the PBS treated group served as the control. The data are mean ± SD, *p* *** **≤** 0.001 vs. the PBS. **(D)** The relative body weight changes in different groups. **(E)** The survival curve in PBS, Ce6, and HA-sese-Ce6 micelles groups. **(F)** Tumor weights of different groups were recorded on the day of natural death or execution (day 40).

To assess the efficient tumor accumulation of HA-sese-Ce6 *via* targeted delivery, the mice bearing 4T1 tumors in the mammary fat pad were injected with free Ce6 and HA-sese-Ce6 micelles *via* tail veins, respectively. *In vivo* Ce6 fluorescence imaging was performed at 1 and 2 h after intravenous injection. As shown in Figure 5B, the accumulation of HA-sese-Ce6 micelles was shown in the liver and cancer cells. This suggested an effective targeted delivery of Ce6 to tumor tissues and the role of the liver in drug clearance. The free Ce6 treated group showed a high liver and kidney accumulation but significant less accumulation of Ce6 in tumors. This indicated a poor drug delivery to tumor tissues and the role of the kidney in drug clearance.

### Anticancer Effect of HA-sese-Ce6 Micelles in Tumor Bearing Animal Model

Figure 5A shows the scheme of the photodynamic therapy in a 4T1 orthotopic mammary fat pad tumor growth model in BALB/c female mice. The day tumor blocks were planted in the mice was considered as day 0; after five times of photodynamic therapy (from day 13 to day 21, every 2 days), survival period of mice was recorded until day 40. In Figure 5C, the HA-sese-Ce6 micelle treated group exhibited the highest anticancer effect (tumor volume on Day25 was similar to the original tumor volume before the treatment), when compared to the free Ce6 treated group (5 fold original tumor volume) and PBS group (10 fold original tumor volume). The tumor growth inhibition effect is likely due to the HA-based target delivery of Ce6 and diselenide-based responsive Ce6 release. Moreover, the mice treated with HA-sese-Ce6 micelles showed the longest survival period among all groups (Figure 5E). This is consistent with the results of the tumor volume change. Tumor weights of the HA-sese-Ce6 treated group were also lighter than those of the free ce6 treated group and the control group, as shown in Figure 5F. In addition, no obvious different in body weight was observed (Figure 5D).

**Figure 6:**
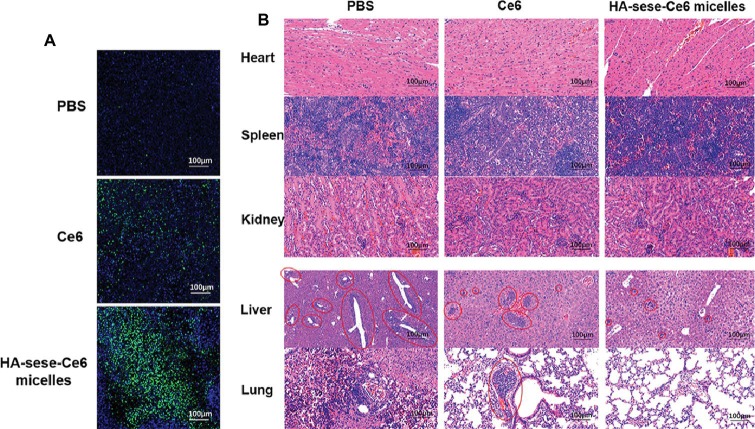
**(A)** TUNEL staining of excised tumor tissues after free Ce6 HA-sese-Ce6 micelles treatments, PBS treatment as control. Apoptotic cells (green, FITC); Normal cancer cells (blue, DAPI). **(B)** H&E staining of heart, spleen, kidney, liver, and lung tissues dissected from mice after different treatments on day 26. Red circles represent tumor metastasis.

To further investigate the effect of HA-sese-Ce6 micelles in promoting apoptosis and inhibiting metastasis, the sections of tumor tissues and other major organs tissues (heart, spleen, kidney, liver, lung) were prepared. TUNEL staining of tumors is shown in Figure 6A, and the greatest number of the apoptosis cells (green) was found in the HA-sese-Ce6 micelles group, when compared to the free Ce6 treated and the PBS treated group. In addition, metastasis in major organs was observed by H&E staining of the heart, spleen, kidney, liver, and lung tissues. The decreased metastasis in the liver and lung was found in the HA-sese-Ce6 micelles treated group (Figure 6B). These results further confirmed that the HA-sese-Ce6 micelles treatment showed a significantly higher anti-cancer effect due to targeted delivery and smart release of Ce6.

## Conclusions

In this study, we developed a minimalist photosensitizer delivery system. HA-sese-Ce6 micelles showed targeted, redox sensitive delivery of Ce6 to 4T1 breast cancer cells. The therapeutic effect of this method could be maximized *via* positive feedback because the activated Ce6 generates singlet oxygen molecules, which helps to break more diselenide bonds on the micelles. These characteristics were confirmed in 4T1 mice breast cancer cells and *in vivo* 4T1 tumor bearing mice models. This unique HA-sese-Ce6 micelles exhibited a great anti-cancer effect and metastasis inhibition. We believe that this can be a promising new strategy for improved photosensitizer delivery in breast cancer treatment.

## Ethics Statement

This study was carried out in accordance with the recommendations of ‘Tongji University Animal Ethics guidelines, name of committee’. The protocol was approved by Tongji University Animal Ethics Committee.

## Author Contributions

CF, CD, and YM designed the experiments. CF, DZ, LC, LL, and JL carried out the experiments. NK, SL, XZ, and YL helped to analyze the experimental results. CF wrote the manuscript.

### Conflict of Interest Statement

The authors declare that the research was conducted in the absence of any commercial or financial relationships that could be construed as a potential conflict of interest.
